# Higher frailty levels are associated with lower cognitive test scores in a multi-country study: evidence from the study on global ageing and adult health

**DOI:** 10.3389/fmed.2023.1166365

**Published:** 2023-06-01

**Authors:** Rosa Estela García-Chanes, José Alberto Avila-Funes, Miguel Germán Borda, Mario Ulises Pérez-Zepeda, Luis Miguel Gutiérrez-Robledo

**Affiliations:** ^1^Dirección de Investigación, Instituto Nacional de Geriatría, Mexico City, Mexico; ^2^Instituto Nacional de Ciencias Médicas y Nutrición Salvador Zubirán, Mexico City, Mexico; ^3^Bordeaux Population Health Research Center, INSERM-University of Bordeaux, UMR 1219, Bordeaux, France; ^4^Centre for Age-Related Medicine (SESAM), Stavanger University Hospital, Stavanger, Norway; ^5^Centro de Investigación en Ciencias de la Salud (CICSA), Facultad de Ciencias de la Salud, Universidad Anáhuac México Campus Norte, Huixquilucan de Degollado, Mexico

**Keywords:** frailty, geriatric epidemiology, cognitive impairment, SAGE, multi-country study

## Abstract

**Background:**

Frailty has been recognized as a growing issue in older adults, with recent evidence showing that this condition heralds several health-related problems, including cognitive decline. The objective of this work is to determine if frailty is associated with cognitive decline among older adults from different countries.

**Methods:**

We analyzed the baseline the Study on Global Ageing and Adult Health (SAGE), that includes six countries (Ghana, South Africa, Mexico, China, Russia, and India). A cross-section analysis was used to assess how Frailty was related with the Clinical Frailty Scale decision tree, while cognitive decline was evaluated using standardized scores of tests used in SAGE.

**Results:**

A total of 30,674 participants aged 50 years or older were included. There was an association between frailty levels and cognitive performance. For example, women had an inverse relationship between frailty levels and cognitive scores, even when comparing robust category with frailty level 2 (RRR = 0.85; *p* = 0.41), although the relative risks decrease significantly at level 3 (RRR = 0.66; *p* = 0.03). When controlling for age, the relative risks between frailty levels 4 to 7 significantly decreased as cognitive performance increased (RRR = 0.46, RRR = 0.52, RRR = 0.44, RRR = 0.32; *p* < 0.001).

**Conclusion:**

Our results show an association between frailty levels measured in a novel way, and cognitive decline across different cultural settings.

## Introduction

Frailty is a common condition that mainly affects older adults and is characterized by the loss of an adequate response to common stressors that leads the individual to a worse health status (1). In fact, global aging has resulted in an increased frequency of conditions such as frailty, increasing the interest from health professionals and researchers in the field of aging and geriatric care (2). Evidence shows that assessing older adults leads to targeted interventions, improving health and especially when focusing this assessment on frailty (3). On the other hand, evidence shows that frailty is closely related to different conditions common to old age (4) and in particular the so-called ‘geriatric syndromes’ (5, 6).

In particular, frailty has been related to deteriorating mental health conditions, such as depression (7), anxiety (8), and cognitive impairment (9). In fact, evidence has grown in recent years, supporting the idea that mental and physical health converge at some point in time, leading to worse health-related outcomes (10, 11), whenever this happens. For example, Avila-Funes et al. showed that frailty is a better predictor of mortality whenever cognitive impairment is considered (12). As previously mentioned, understanding the underpinnings of this relationship has the potential of designing interventions that improve both frailty and cognitive performance in old age (13).

Finally, there is increasing data on how social determinants of health, impact the development and expression of frailty and dementia; those older adults who live in countries with high inequality have the most adverse outcomes (14–18). Taking advantage of the multi-country design of the Study on Global Ageing and Adult Health (SAGE) (includes six low- and middle-income countries) we aimed at understanding the association between frailty and cognitive performance in older adults from different sociocultural background.

## Methods

### Design and sample

A cross-sectional was used to baseline data of SAGE (2007–2010). This study was an initiative from the World Health Organization (WHO) to determine the factors that impact aging in six low- and middle-income countries (LMICs): Ghana, Mexico, India, South Africa, Russia, and China. It includes a set of harmonized questionnaires from different dominions (e.g., health, physical function, demographics, etc.), which was applied to a representative sample of adults aged 50 or older from the participant countries. Complete description of objectives and procedures is available elsewhere (19). For purposes of this report, only participants with complete baseline information from all the countries were included.

Ethical approval. The SAGE study was approved by the World Health Organization’s Ethical Review Board (reference number RPC149) and the Ethical and Protocol Review Committee of each country. Written informed consent was obtained from all study respondents.

### Clinical Frailty Scale Decision Tree

Currently, numerous tools have been developed and used to categorize someone as frail (20). Among these instruments, the Clinical Frailty Scale, was developed to derive frailty levels after a geriatric assessment, resulting in scores from 1 (robust older adult) to 9 (end-stage frailty) (21). Using figurines and descriptive test for each level of frailty, the trained clinician can derive a category, that in turn may trigger interventions (22). Recently, a decision tree was developed to facilitate the application of the Clinical Frailty Scale (CFS) (21) for a wider range of professionals, not necessarily experts in the field (23). Since this is a very recent add-on to CFS, reports on its usage are still limited. However, a large study showed its ability to predict adverse health-related outcomes in older adults admitted in the intensive care unit (24), so its application in the community could be very useful.

**Figure 1:**
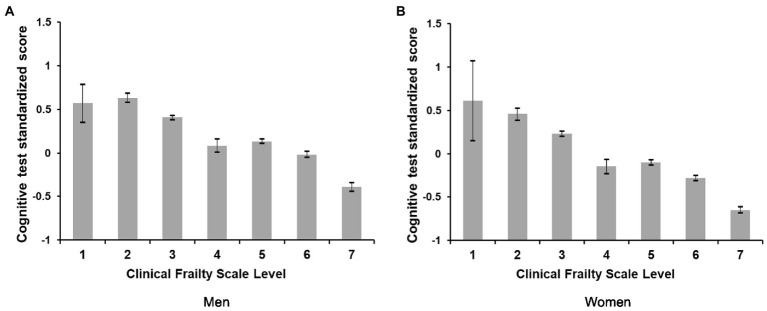
Comparison of the cognitive tests standardized scores between Clinical Frailty Scale levels by sex.

As with the CFS, the decision tree results in frailty levels. The levels of the CFS decision tree were integrated according to instructions available in the original manuscript (23) and the website.^1^ The classification of frailty levels was built from the variables available in the SAGE survey including: activities of daily living (ADL)—bathing, getting dressed, eating, walking, getting up; instrumental ADL (IADL)—housework, participating in community activities, doing your day to day work, carrying things, using private or public transport, getting out of your home; chronic diseases—arthritis, stroke, angina, diabetes, chronic lung disease, asthma, depression, hypertension, cataract; self-rated health; fatigue—you have enough energy for everyday life; physical activity—any moderate-intensity sports, fitness or leisure activities causing a small increase in breathing or heart rate for at least 10 min at a time (See Supplementary Table A and Figure 1). Using the classification tree, frailty levels were defined from 1 to 7 according to the flow of conditions and characteristics reported by older persons. Levels 8 and 9 refer to individuals with terminal conditions, since SAGE does not include them, these levels were not considered.

### Cognitive performance

The SAGE includes five cognitive tests to assess this dominion: immediate and delayed verbal recall (i.e., episodic memory), forward and backward digit span (i.e., working memory), and verbal fluency (i.e., semantic memory). For the immediate verbal recall test, interviewers first read a list of 10 words aloud and asked the participants to immediately recall as many words as they could in one minute (three trials), recording the best score. The delayed recall ability was assessed by asking participants to recall the list of words; and scored in a similar fashion as the previous test. Forward and backward digit span, scoring the number of correct series repeating in two trials (9 points-forward span and 8 points-backward span). Naming as many animals that come to one’s mind in one minute was used to test verbal fluency (spontaneous language production), with possible scores ranging from 0 to 70 (one point for each valid animal name). *Z*-scores were calculated to facilitate the comparison of the cognitive tests’ performance, between individuals and countries; a previously reported method (25). Furthermore, individual *z*-scores from each cognitive domain were added to obtain a global composite *z*-score for cognitive performance by countries.

### Statistical analysis

Initially, all variables included were described by sex. Differences in the means for the composite cognitive *z*-score between CFS levels—also by sex—was done with one-way ANOVA, including *post hoc* estimation with Bonferroni’s test. The analysis of the CFC levels and the z scores of each one of the cognitive domains is also presented (see Supplementary Table B). The descriptive and ANOVA analyzes were also done for each country (see Supplementary Tables C, D). Finally, three multinomial models were fitted by sex to estimate the effects of age and education on cognition between CFS levels and their corresponding estimated probabilities were plotted. Additionally, adjusted models for country were performed (see Supplementary Table E). All statistical analyses were performed with STATA version 17.0.

## Results

From the 35,330 participants aged 50 or older, 30,674 (86.8%) had complete data on the variables of interest. Our study sample was constituted by participants from China (39.5%), Ghana (13.1%), India (20.4%), México (6.8%), Russia (10.6%), and South Africa (9.6%) (See Supplementary Table C). When analyzing the total sample by sex, no differences in age were observed, but there was higher level of education in men.

Regarding CFS levels, higher levels of frailty were more frequent for women (26.5% in level 6 and 14.5% in level 7). These differences by sex were clear when observed in the number of chronic diseases (0 men vs. 1 women), in worse health perception (15.7% in men vs. 19.8% in women), physical activity (13.7% in men vs. 10.6% women) (see Table 1). An inverse relationship was found between the cognitive *z*-score and levels of frailty and sex, 1 ( $\bar{x}$ men = 0.57,95%CI [0.35to0.79]; $\bar{x}$ women = 0.61,95%CI[0.15to1.08]), level 2 ( $\bar{x}$ men = 0.63,95%CI[0.58to0.69]); $\bar{x}$ women = 0.46,95%CI[0.40to0.53]), level 3 $\bar{x}$ men = 0.41,95%CI[0.38to 0.43]); $\bar{x}$ women = 0.23,95%CI[0.20 to 0.26]), level 4 ( $\bar{x}$ men = 0.08,95%CI[−0.01to 0.16]; $\bar{x}$ women −.15,95%CI[−0.23to−0.62]), level 5 ( $\bar{x}$ men = 0.13,95%CI[0.11to 0.16]; $\bar{x}$ women = −0.10,95%CI[−0.13to−0.07]) level 6 $\bar{x}$ men = −0.02,95%CI [−.05to0.02]); $\bar{x}$ women = −0.28,95%CI[−0.31to−0.25]), level 7 $\bar{x}$ men = −0.39,95%CI[−0.44to−0.34]); $\bar{x}$ women = −0.65,95%CI[−0.68to−0.60]), (see Figure 1). When analyzing the differences for the composite cognitive *z*-score between levels of frailty, no significant differences were found for levels below 4. Moreover, there were no differences in the cognitive score between levels 4 and 5 in both men and women (see Table 2 and Figure 1).

**Table 1:** Descriptive statistics stratified by sex.

Variables	Total (*n* = 30,674)	Male (*n* = 14,060)	Female (*n* = 16,614)
Age, mean (SD)	63.30 (9.60)	63.20 (9.50)	63.4 (9.70)*
Education, mean (SD)	5.50 (5.00)	6.30 (5.00)	4.8 (5.00)**
Clinical Frailty Scale, *n* (%)			
1	89 (0.30)	60 (0.40)	29 (0.20)*
2	1,906 (6.20)	1,107 (7.90)	799 (4.80)*
3	7,472 (24.40)	3,904 (27.80)	3,568 (21.50)**
4	941 (3.10)	431 (3.10)	510 (3.10)
5	8,861 (28.90)	3,966 (28.20)	4,895 (29.50)
6	7,405 (24.10)	2,995 (21.30)	4,410 (26.50)**
7	4,000 (13.00)	1,597 (11.40)	2,403 (14.50)*
ADL, median (IQR)	0 (1.00)	0 (1.00)	0 (1.00)**
IADL, median (IQR)	1 (4.00)	1 (3.00)	2 (4.00)**
Number of chronic diseases, median (IQR)	1 (2.0)	0 (1.0)	1 (2.0)**
SRH, *n* (%)			
Very good	893 (2.9)	533 (3.8)	360 (2.2)
Good	8,892 (29.0)	4,650 (33.1)	4,242 (25.5)**
Moderate	14,833 (48.4)	6,462 (46.0)	8,371 (50.4)**
Bad	5,493 (17.9)	2,206 (15.7)	3,287 (19.8)**
Very bad	563 (1.8)	209 (1.5)	354 (2.1)
Energy, *n* (%)	5,328 (17.4)	2,748 (19.5)	2,580 (15.5)**
Physical activity, *n* (%)	3,693 (12.1)	1,929 (13.7)	1,764 (10.6)*
Cognitive tests standardized score, mean (SD)	0.01 (0.99)	0.15 (0.98)	−0.13 (0.98) **
Immediate verbal Recall, mean (SD)	4.7 (1.7)	4.8 (1.8)	4.6 (1.7) **
Forward digit span, mean (SD)	5.6 (1.8)	5.8 (1.8)	5.5 (1.8) **
Backward digit span, mean (SD)	3.0(1.6)	3.2 (1.5)	2.8 (1.6) **
Verbal Fluency, mean (SD)	12.2 (5.0)	12.7 (5.0)	11.8 (4.9) **
Delayed verbal Recall, mean (SD)	4.9 (2.2)	5.0 (2.2)	4.8 (2.2) **

ADL, activities of daily living; IADL, instrumental activities of daily living; SRH, self-rated health. Test of means and proportions by sex *p < 0.005 and **p < 0.001.

**Table 2:** Composite cognitive *z*-scores mean differences by Clinical Frailty Scale Level and stratified by sex, including *post hoc* Bonferroni’s test.

CFS level	Male						Female					
	1	2	3	4	5	6	1	2	3	4	5	6
2	0.06	1	–	–	–	–	−0.14	1	–	–	–	–
3	−0.16	−0.22	1	–	–	–	−0.38	−0.23**	1	–	–	–
4	−0.49*	−0.55**	−0.33*	1	–	–	−0.75*	−0.60**	−0.37**	1	–	–
5	−0.43**	−0.49**	−0.27**	0.05	1	–	−0.71*	−0.56*	−0.33**	0.04	1	–
6	−0.58**	−0.65**	−0.42**	−0.09	−0.15*	1	−0.89**	−0.74**	−0.50**	−0.13*	−0.17**	1
7	−0.95**	−1.02**	−0.79**	−0.46**	−0.52**	−0.37**	−1.25**	−1.10**	−0.87**	−0.50**	−0.54**	−0.36**

CFS, Clinical Frailty Scale. *p < 0.005 and **p < 0.001.

Table 3 shows the relative risk ratios (RRR) obtained from the unadjusted and adjusted multivariable multinomial logistic regression models that differentiated the cognitive score between frailty levels with level 1 (Very Fit) as the reference category. For men, the unadjusted model showed that for each additional unit of the cognitive score, the relative risks of being fit (level 2) (RRR = 1.08; *p* = 0.60) compared to being level 1 did not increase significantly. On the other hand, the risks related to having very mild (level 4), mild (level 5), moderate (level 6), and severe frailty (level 7) decreased significantly (RRR = 0.57, RRR = 0.61, RRR = 0.51, RRR = 0.34; *p* < 0.001) as cognitive scores increased. When controlling for age (model 2), this inverse association between cognition and frailty levels was maintained (RRR = 0.56, RRR = 0.66, RRR = 0.58, RRR = 0.41; *p* < 0.001). Furthermore, when adjusting for education and age, the differences between the cognitive score and the CFS levels were reduced; and when comparing level 5 to level 1 lost significance (RRR = 0.80; *p* = 0.13).

**Table 3:** Sex-stratified multinomial regression models for composite cognitive *z*-score and Clinical Frailty Scale level (level 1 as reference).

CFS level	Men				Women			
	Model 1*	Model 2†	Model 3 ∫	Model 3∫*	Model 1 *	Model 2 †	Model 3 ∫	Model 3 ∫*
	(RRR, 95% CI, value of *p*)							
1	Reference level							
2	1.08 (0.82–1.42, 0.60)	1.06 (0.80–1.41, 0.68)	1.13 (0.84–1.53, 0.42)	1.16 (0.85–1.59, 0.346)	0.85 (0.58–1.25, 0.41)	0.90 (0.60–1.34, 0.60)	0.98 (0.63–1.53, 0.94)	0.98 (0.63–1.54, 0.933)
3	0.83 (0.63–1.09, 0.18)	0.81 (0.61–1.07, 0.14)	0.95 (0.70–1.28, 0.72)	1.03 (0.76–1.40, 0.861)	0.66 (0.45–0.96, 0.03)	0.68 (0.46–1.01, 0.06)	0.77 (0.50–1.19, 0.24)	0.79 (0.51–1.22, 0.288)
4	0.57 (0.43–0.76, <0.001)	0.56 (0.41–0.75, <0.001)	0.66 (0.48–0.90, 0.01)	0.72 (0.52–1.00, 0.049)	0.43 (0.29–0.64, <0.001)	0.46 (0.31–0.69, <0.001)	0.56 (0.35–0.87, 0.01)	0.58 (0.37–0.92, 0.20)
5	0.61 (0.46–0.80, <0.001)	0.66 (0.50–0.87, <0.001)	0.80 (0.59–1.07, 0.13)	0.77 (0.57–1.05, 0.097)	0.45 (0.31–0.66, <0.001)	0.52 (0.35–0.77, 0.001)	0.65 (0.42–1.01, 0.05)	0.64 (0.41–1.00, 0.049)
6	0.51 (0.39–0.68, <0.001)	0.58 (0.47–0.77, <0.001)	0.74 (0.55–0.99, 0.04)	0.68 (0.50–0.92, 0. 014)	0.37 (0.25–0.54, <0.001)	0.44 (0.30–0.65, <0.001)	0.57 (0.36–0.88, 0.01)	0.64 (0.41–1.00, 0.49)
7	0.34 (0.26–0.45, <0.001)	0.41 (0.31–0.55, <0.001)	0.51 (0.38–0.69, <0.001)	0.44 (0.32–0.60, <0.001)	0.24 (0.17–0.36, <0.001)	0.32 (0.22–0.48, <0.001)	0.40 (0.26–0.62, <0.001)	0.55(0.35–0.85, 0.08)

CFS, Clinical Frailty Scale; RRR, relative risk ratio; CI, confidence interval. ^*^Not adjusted. ^†^Adjusted for age. ∫Adjusted for age and education. ∫*Adjusted for age, education and country.

For women, the inverse relationship between frailty levels was maintained as the cognitive score increases even for level 2 (RRR = 0.85; *p* = 0.41), although the relative risks decreased significantly at level 3 (RRR = 0.66; *p* = 0.03). When controlling for age, the relative risks between levels 4 and 7 significantly decrease as cognitive performance increases (RRR = 0.46, RRR = 0.52, RRR = 0.44, RRR = 0.24; *p* < 0.001) (see Table 3).

**Figure 2:**
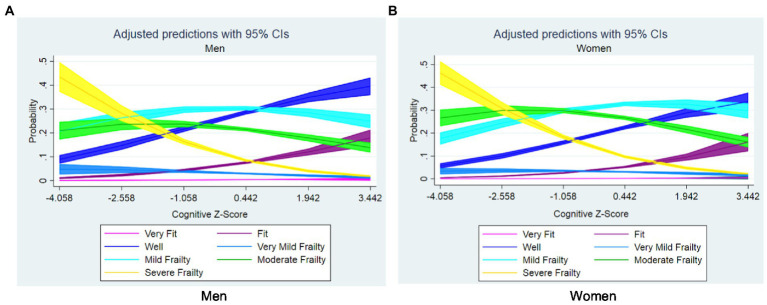
Sex-stratified estimated probabilities of frailty levels according to composite cognitive *z*-score, adjusted for age and education: **(A)** men and **(B)** women.

Finally, regarding the sex-stratified predicted probabilities for cognitive performance by different levels of frailty (see Figure 2), the probability to experience higher levels of frailty (i.e., CFS 7) increases with lowering cognitive performance. Moreover, having lower frailty levels (i.e., CFS 2 or 3) decreases the probability of having cognitive impairment.

## Discussion

To the best of our knowledge this is the first study to assess the association between cognitive performance tests and frailty measured using the CFS decision tree. Moreover, this adds to the current knowledge on how frailty relates to cognitive status in older adults, the higher the burden of frailty the lower the cognitive test scores. As shown in our results, those in charge of older adults should be careful to further examinate the cognitive status in those individuals with 3 or higher levels. Considering the CFS is a preliminary evaluation that leads to a more comprehensive assessment, the decision tree also showed that these levels are not limited to a progression in frailty, but alert that there might be a global decay in the older adult (i.e., mental and physical health), that merits further assessment (23, 26).

Previous evidence shows that frailty could precede dementia and other neurocognitive disorders, and even be related to neuropathological findings (27–29). On the other hand, there have been some interventions—mainly based in physical activity—that have shown to improve the overall health status of an older adult with frailty (30, 31), and this has the potential to stop the progression of both cognitive and physical decline. However, this relationship is still not fully understood, and merits further research.

Interestingly, our results were from a multi-national sample—the SAGE study—diming the effect of cross-cultural issues when assessing, since it utilizes a common tool and standardization with *z*-scores was used in this work, similar to what was used previously (25, 32). This points to the possible true nature of the association of mental and physical health, regardless of sociocultural differences. Assessment with the CFS decision tree allows to have a quick screening of an individual, as a screening of global health, and according to our results it could also alert about mental health. For instance, older adults with CFS level 3 or lower have the lowest probability of having low cognitive test scores and might not need further evaluation. In contrast, individuals with CFS levels ≥4 might benefit from a further evaluation, possibly focusing on cognition.

Although the tree is recommended only as an aid in assessing an older adult, it could be useful as a screen measure in an incremental approach to assessing older adults. Other studies have shown similar results, using other tools to measure frailty and other cognitive tests, whilst results seem consistent with that previously reported (12, 33–37). Moreover, one of the advantages that the CFS has is the ability to capture a continuum of frailty, rather than a limited ‘all or nothing’ grouping that almost all the other tools used for assessing frailty provide. This is helpful since interventions or different levels of care can be tailored to each of the levels. Moreover, a combination of CFS level with the presence or not of cognitive impairment is an add-on.

It is well-known that older population continues to grow, but that there are not enough resources to attend the needs of this age group. The CFS decision tree, could aid in identifying those at risk not only of increased risk of adverse health-related outcomes (23, 38, 39), but also of those that could be in the verge of presenting overt dementia, and benefit from early intervention for frailty that could impact cognitive health.

We need to acknowledge the limitations of our work. First, as a cross-sectional study, is difficult to address causality; though, longitudinal studies have shown that this might be the case. The SAGE has advantages, but there is no availability of follow-up (only for Mexico, currently), and some years have passed since the baseline data was released. Cognitive status was not assessed with the state-of-the-art evaluation, but similar approaches to testing cognition have been used in epidemiologic studies. Finally, we recognize that having a multi-country approach could be in one hand, an advantage, but in the other also a source of variability. Further research using these data (and hopefully follow-ups) should be aimed at unveiling the relationships of social, cultural, and economic characteristics of each country when it comes to the relationship between frailty and cognition.

## Conclusion

The CFS decision tree used to assess frailty shows differences with lower scores for cognitive tests across different countries, adding to the existing knowledge an easy-to-use tool to assess frailty on how cognition and physical health are closely related.

## Data availability statement

The raw data supporting the conclusions of this article will be made available by the authors, without undue reservation. The datasets are available in the WHO repository, http://apps.who.int/healthinfo/systems/surveydata/index.php/catalog/sage/about.

## Author contributions

RG-C performed the statistical analysis and wrote the first draft. JA-F and MB reviewed and added additional ideas. MP-Z had the original idea and wrote the first draft and added additional ideas for the statistical analysis. LG-R supervised all the stages of the manuscript and added additional ideas. All authors contributed to the article and approved the submitted version.

## Funding

The publication of this paper was supported by Instituto Nacional de Geriatría, México.

## Conflict of interest

The authors declare that the research was conducted in the absence of any commercial or financial relationships that could be construed as a potential conflict of interest.

## Publisher’s note

All claims expressed in this article are solely those of the authors and do not necessarily represent those of their affiliated organizations, or those of the publisher, the editors and the reviewers. Any product that may be evaluated in this article, or claim that may be made by its manufacturer, is not guaranteed or endorsed by the publisher.

## Supplementary material

The Supplementary material for this article can be found online at: https://www.frontiersin.org/articles/10.3389/fmed.2023.1166365/full#supplementary-material

## Footnotes

^1^ https://www.dal.ca/sites/gmr/our-tools/clinical-frailty-scale/cfs-classification-tree.html
