# Supplementary material for: Higher frailty levels are associated with lower cognitive test scores in a multi-country study: evidence from the study on global ageing and adult health
Source: Front Med (Lausanne). 2023 Jun 1;10:1166365. doi: 10.3389/fmed.2023.1166365 (PMC10267459; doi:10.3389/fmed.2023.1166365)
Supplement: Supplementary file 1 [file Data_Sheet_1.docx]

**Supplement**

Table A. Variables included for the Clinical Frailty Scale decision tree

| Category | Question | | Answer options | Decision Variables Tree |
| --- | --- | --- | --- | --- |
| Activities of daily living (BADLs) | In the last 30 days, how much difficulty did you have… | Bathing/washing, getting dressed, eating, walking, getting up from lying down | None  Mild  Moderate  Severe  Extreme/cannot do | 0 (BALDs)  3-5 (BALDs)  1-2 (BALDs) |
| Instrumental activities of daily living (IADLs) |  | Taking care of your household responsibilities, joining in community activities, doing your day-to-day work, with carrying things, using private or public transport, getting out of your home |  | 0 (IADLs)  1-4 (IADLs)  5-6 IADLs |
| Chronic diseases | Have you ever been diagnosed with/told you have… | Arthritis, stroke, angina, diabetes, chronic lung disease, asthma, depression, hypertension, cataract | Yes  No | 0-9 (Chronic diseases) |
| Self-rated health | In general, how would you rate your health today? | | Very good  Good  Moderate  Bad  Very bad | 1 (Very good)  2 (Good /Moderate)  3 (Bad / Very Bad) |
| Effort | Do you have enough energy for everyday life? | | Completely  Mostly  Moderately  A little  Not at all | 1 Completely  2 (Mostly, Moderately)  3 (A little  None) |
| Physical activity | Do you do any moderate-intensity sports, fitness or recreational (leisure) activities that causes a small increase in breathing or heart rate for at least 10 minutes at a time? | | Yes  No | Yes  No |

Figure 1. Clinical Frailty Scale (CFS) adapted for SAGE


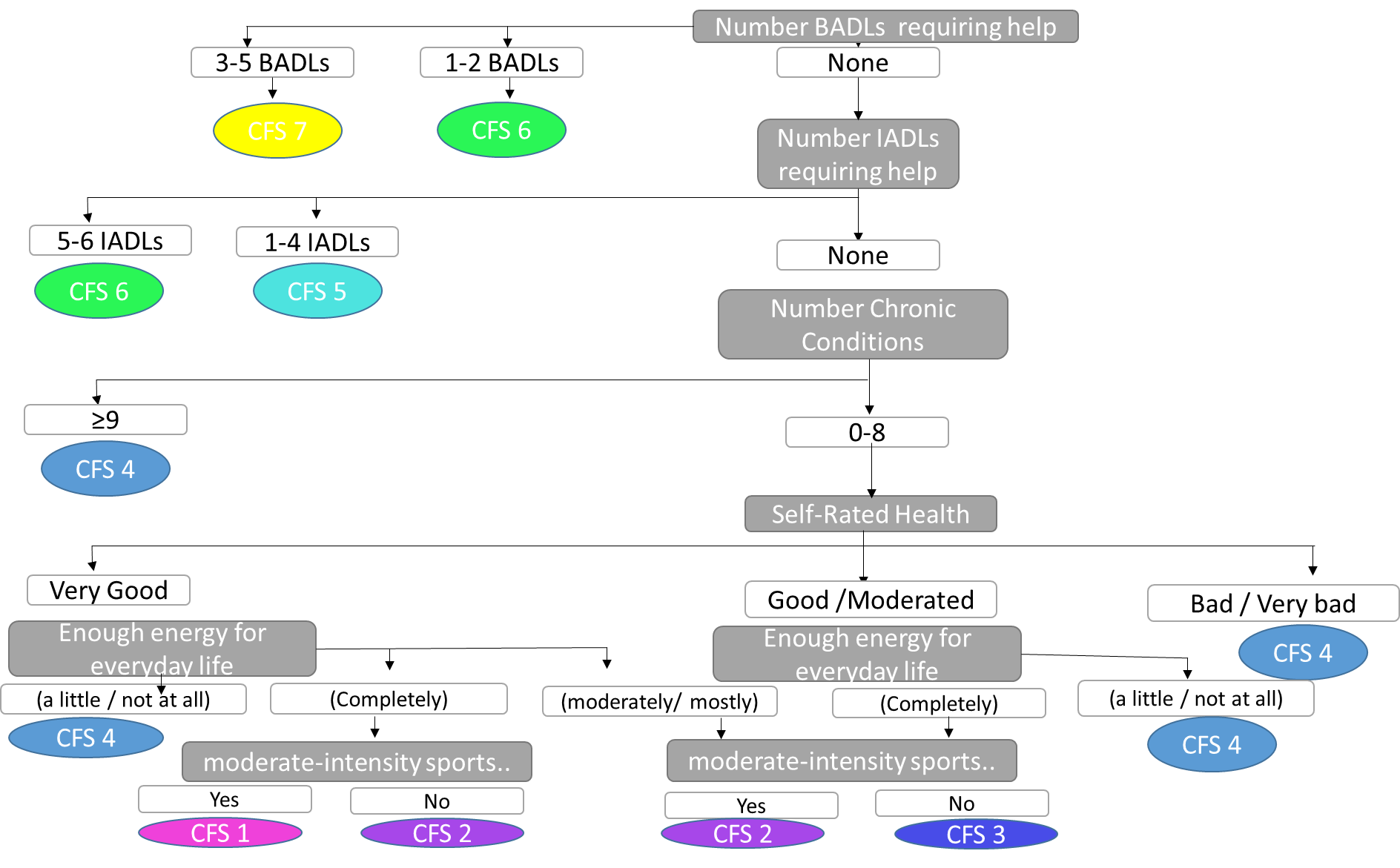


Table B. Each cognitive domains z-scores mean differences by Clinical Frailty Scale Level and stratified by sex, including *post hoc* Bonferroni´s test.

| Immediate verbal Recall | | | | | | | | | | | | |
| --- | --- | --- | --- | --- | --- | --- | --- | --- | --- | --- | --- | --- |
| CFS level | Male | | | | | | Female | | | | | |
|  | 1 | 2 | 3 | 4 | 5 | 6 | 1 | 2 | 3 | 4 | 5 | 6 |
| 2 | 0.14 | 1 | - | - | - | - | 0.15 | 1 | - | - | - | - |
| 3 | 0.08 | -0.06 | 1 | - | - | - | 0.06 | -0.09 | 1 | - | - | - |
| 4 | -0.24 | -0.38** | -0.32** | 1 | - | - | -0.26 | -0.40** | -0.32** | 1 | - | - |
| 5 | -0.11 | -0.24** | -0.19** | 0.14 | 1 | - | -0.14 | -0.29** | -0.20** | 0.12 | 1 | - |
| 6 | -0.25 | -0.39** | -0.33** | -0.01 | -0.14** | 1 | -0.29 | -0.44** | -0.35** | -0.03 | -0.15** | 1 |
| 7 | -0.40* | -0.53** | -0.48** | -0.15 | -0.29** | -0.15** | -0.51 | -0.66** | -0.57** | -0.25** | -0.37** | -0.22** |
| Forward digit span | | | | | | | | | | | | |
| 2 | -0.14 | 1 |  |  |  |  | -0.13 | 1 |  |  |  |  |
| 3 | -0.28 | -0.15** | 1 |  |  |  | -0.30 | -0.17** | 1 |  |  |  |
| 4 | -0.46* | -0.33** | -0.18* | 1 |  |  | -0.50* | -0.37** | -0.20** | 1 |  |  |
| 5 | -0.48** | -0.35** | -0.20** | -0.02 | 1 |  | -0.58* | -0.45** | -0.28** | -0.08 | 1 |  |
| 6 | -0.52** | -0.39** | -0.24** | -0.06 | -0.04 | 1 | -0.62* | -0.49** | -0.32** | -0.12* | -0.04 | 1 |
| 7 | -0.80** | -0.67** | -0.52** | -0.34** | -0.32** | -0.28** | -0.81** | -0.68** | -0.51** | -0.31** | -0.23** | -0.19** |
| Backward digit span | | | | | | | | | | | | |
| 2 | 0.00 |  |  |  |  |  | -0.34 | 1 |  |  |  |  |
| 3 | -0.16 | -0.16** |  |  |  |  | -0.47 | -0.13* | 1 |  |  |  |
| 4 | -0.46* | -0.46** | -0.30** |  |  |  | -0.78** | -0.44** | -0.31** | 1 |  |  |
| 5 | -0.32 | -0.33** | -0.16** | 0.14 |  |  | -0.74* | -0.41** | -0.27** | 0.03 | 1 |  |
| 6 | -0.48* | -0.48** | -0.31** | -0.01 | -0.15** |  | -0.88** | -0.54** | -0.41** | -0.1 | -0.13** | 1 |
| 7 | -0.64** | -0.64** | -0.48** | -0.18* | -0.32** | -0.17** | -1.04** | -0.70** | -0.57** | -0.26** | -0.29** | -0.16** |
| Verbal Fluency | | | | | | | | | | | | |
| 2 | 0.11 | 1 |  |  |  |  | 0.03 |  |  |  |  |  |
| 3 | -0.16 | -0.27** | 1 |  |  |  | -0.23 | -0.26** |  |  |  |  |
| 4 | -0.28 | -0.39** | -0.12 | 1 |  |  | -0.39 | -0.42** | -0.16* |  |  |  |
| 5 | -0.31 | -0.41** | -0.15** | -0.03 | 1 |  | -0.35 | -0.38** | -0.12** | 0.04 |  |  |
| 6 | -0.35 | -0.45** | -0.18** | -0.06 | -0.04 | 1 | -0.43 | -0.46** | -0.2** | -0.04 | -0.08* |  |
| 7 | -0.74** | -0.85** | -0.58** | -0.46** | -0.44** | -0.40** | -0.76** | -0.79** | -0.53** | -0.37** | -0.41** | -0.33** |
| Delayed verbal Recall | | | | | | | | | | | | |
| 2 | 0.13 |  |  |  |  |  | -0.21 |  |  |  |  |  |
| 3 | -0.00 | -0.13* |  |  |  |  | -0.34 | -0.14* |  |  |  |  |
| 4 | -0.23 | -0.36** | -0.23** |  |  |  | -0.66* | -0.45** | -0.31** |  |  |  |
| 5 | -0.24 | -0.37** | -0.24** | -0.01 |  |  | -0.60* | -0.40** | -0.26** | 0.05 |  |  |
| 6 | -0.32 | -0.45** | -0.32** | -0.09 | -0.08 |  | -0.73* | -0.52** | -0.38** | -0.07 | -0.12** |  |
| 7 | -0.57** | -0.70** | -0.57** | -0.34** | -0.33** | -0.25** | -1.01** | -0.81** | -0.67** | -0.35** | -0.41** | -0.29** |

CFS=Clinical Frailty Scale

*p<0.005

** p<0.001

Table C. Descriptive statistics of main variables stratified by country and sex

| Variables | Countries (Total) | | | | | |
| --- | --- | --- | --- | --- | --- | --- |
|  | China (n=12,115) | Ghana (n=4,030) | India (n=6,261) | Mexico (n=2,069) | Russia (n=3,258) | South Africa (n=2,941) |
| Age, mean (SD) | 63.0 (9.3) | 64.1 (10.5)^+^ | 61.6 (8.8) | 68.8 (9) | 64.5 (10.0) ^+^ | 62.7 (9.5) |
| Women, n % | 6,475 (53.5) | 1,917 (47.6) | 3,065(49.0) | 1,258 (60.8) | 2,118 (65.0) | 1,781(60.6) |
| Education, mean (SD) | 5.4(4.5)+ | 4.2(5.3)+ | 3.7(4.8) | 4.5(4.2)+ | 11.1(3.8) | 5.8(4.7) |
| Cognitive tests standardized scores, mean (SD) | -0.005(1.002) | -0.006(0.998) | 0.001(1.000) | 0.073(0.916) | 0.012(0.999) | -0.023(0.997) |
| Clinical Frailty Scale, n(%) |  |  |  |  |  |  |
| 1 | 48 (0.4) | 7 (0.2) | 9 (0.1) | 6 (0.3) | 3 (0.1) | 7 (0.5) |
| 2 | 1,124 (9.3) | 274 (6.8) | 196 (3.1) | 47 (2.3) | 141 (4.3) | 274 (4.2) |
| 3 | 4,153 (34.3) | 542 (13.5) | 535 (8.5) | 405 (19.6) | 781 (24.0) | 542 (35.9) |
| 4 | 603 (5.0) | 51 (1.3) | 44 (0.7) | 27 (1.3) | 62 (1.9) | 51 (5.2) |
| 5 | 4,054 (33.5) | 1,371 (34.0) | 1,686 (26.9) | 547 (26.4) | 677 (20.8) | 1,371 (17.9) |
| 6 | 1,510 (12.5) | 1,079 (26.8) | 2,607 (41.6) | 561 (27.1) | 953 (29.3) | 1,079 (23.6) |
| 7 | 623 (5.1) | 706 (17.5) | 1,184 (18.9) | 476 (23.0) | 641 (19.7) | 706 (12.6) |
| ADL, median (IQR) | 0(0-0) | 0(0-1)+ | 1(0-2)+ | 0(0-2)+ | 0(0-2)+ | 0(0-1) |
| IADL, median (IQR) | 0(0-2) | 2(1-4) | 3(1-5) | 2(0-4)+ | 2(0-4)+ | 1(0-3) |
| Number of chronic diseases, median (IQR) | 1(0-1)+ | 0(0-1)+ | 1(0-1)+ | 1(0-2) | 2(1-3) | 1(0-1)+ |
| SRH, n (%) |  |  |  |  |  |  |
| Very good | 393 (3.2) | 166 (4.1) | 123 (2.0) | 55 (2.7) | 16 (0.5) | 140(4.8) |
| Good | 3,657 (30.2) | 1,489 (37.0) | 1,633 (26.1) | 708 (34.2) | 401 (12.3) | 1,004 (34.1) |
| Moderate | 5,534 (45.7) | 1,731 (43.0) | 3,237(51.7) | 1,038 (50.2) | 1,960 (60.2) | 1,333 (45.3) |
| Bad | 2,275 (18.8) | 563 (14.0) | 1,160 (18.5) | 254 (12.3) | 824 (25.3) | 417 (14.2) |
| Very bad | 256 (2.1) | 81 (2.0) | 108 (1.7) | 14 (0.7) | 57 (1.8) | 47 (1.6) |
| Energy, n (%) | 3,149 (26.0) | 220 (5.5) | 450(7.2) | 506 (24.5) | 715 (22.0) | 228(9.8) |
| Physical activity, n (%) | 1,806 (5.0) | 558(13.9) | 772(11.5) | 115(5.6) | 311(10.0) | 181(6.2) |

+ No significant differences were found between these countries.

| Variables | Countries (Men) | | | | | |
| --- | --- | --- | --- | --- | --- | --- |
|  | China (n=5,640) | Ghana (n=2,113) | India (n=3,196) | Mexico (n=811) | Russia (n=1,140) | South Africa (n=1,160) |
| Age, mean (SD) | 63.1 (9.3)+ | 63.5 (10.3)+ | 62.3 (8.9)+ | 68.0 (9.2) | 63.3 (9.7)+ | 62.1 (9.1)+ |
| Education, mean (SD) | 6.5 (4.2)+ | 5.4 (5.7)+ | 5.4 (5.1)+ | 4.8 (4.3) | 11.4 (3.6) | 6.1 (4.9)+ |
| Cognitive tests standardized scores, mean (SD) | 0.114 (0.988) | 0.220 (0.994) | 0.260 (0.969) | 0.097 (0.916) | 0.060 (0.996) | 0.110 (0.976) |
| Clinical Frailty Scale, n(%) |  |  |  |  |  |  |
| 1 | \| 31 (0.6) \| 4 \| 2 \| 9 \| \| --- \| --- \| --- \| --- \| | 5 (0.2) | 9 (0.3) | 4 (0.5) | 2 (0.2) | 9 (0.8) |
| 2 | 601 (10.7) | 189 (8.9) | 153 (4.8) | 23 (2.8) | 71 (6.2) | 70 (6.0) |
| 3 | 2,179 (38.6) | 351 (16.6) | 349 (10.9) | 212 (26.1) | 346 (30.4) | 467 (40.3) |
| 4 | 286 (5.1) | 25 (1.2) | 29 (0.9) | 16 (2.0) | 26 (2.3) | 49 (4.2) |
| 5 | 1,668 (29.6) | 726 (34.4) | 956 (29.9) | 202 (24.9) | 235 (20.6) | 179 (15.4) |
| 6 | 621 (11.0) | 466 (22.1) | 1,173 (36.7) | 183 (22.6) | 298 (26.1) | 254 (21.9) |
| 7 | 254 (4.5) | 351 (16.6) | 527 (16.5) | 171 (21.1) | 162 (14.2) | 132 (11.4) |
| ADL, median (IQR) | 0 (0-0) | 0 (0-1)+ | 0 (0-2)+ | 0 (0-2)+ | 0 (0-1)+ | 0 (0-1)+ |
| IADL, median (IQR) | 0 (0-1) | 2 (0-4) | 3 (1-5) | 1 (0-4)+ | 1 (0-4)+ | 0 (0-3) |
| Number of chronic diseases, median (IQR) | 0 (0-1)+ | 0 (0-1) | 1 (0-1)+ | 1 (0-1)+ | 1 (0-2) | 0 (0-1) |
| SRH, n (%) |  |  |  |  |  |  |
| Very good | 223 (4.0) | 114 (5.4) | 82 (2.6) | 31 (3.8) | 11 (1.0) | 72 (6.2) |
| Good | 1,886 (33.4) | 864 (40.9) | 971 (30.4) | 296 (36.5) | 194 (17.0) | 439 (37.8) |
| Moderate | 2,497 (44.3) | 853 (40.4) | 1,567 (49.0) | 397 (49.0) | 682 (59.8) | 466 (40.2) |
| Bad | 941 (16.7) | 248 (11.7) | 532 (16.7) | 79 (9.7) | 245 (21.5) | 161 (13.9) |
| Very bad | 93 (1.7) | 34 (1.6) | 44 (1.4) | 8 (1.0) | 8 (0.7) | 22 (1.9) |
| Energy, n (%) | 1,622 (28.8) | 157 (7.4) | 294 (9.2) | 206 (25.4) | 327 (28.7) | 142 (12.2) |
| Physical activity, n (%) | 870 (15.5) | 331 (15.7) | 463 (14.5) | 50 (6.2) | 129 (11.3) | 86 (7.4) |

+ No significant differences were found between these countries.

| Variables | Countries (Women) | | | | | |
| --- | --- | --- | --- | --- | --- | --- |
|  | China (n=6,475) | Ghana (n=1,917) | India (n=3,065) | Mexico (n=1,258) | Russia (n=2,118) | South Africa (n=1,781) |
| Age, mean (SD) | 62.9 (9.3)+ | 64.8 (10.6)+ | 60.9 (8.7) | 67.8 (9.0) | 65.2 (10.2)+ | 63.0 (9.8)+ |
| Education, mean (SD) | 5 (5)+ | 2.9 (4.6) | 2 (3.6) | 4 (4)+ | 11 (3.9) | 5.5 (4.6) |
| Cognitive tests standardized scores, mean (SD) | -0.109(1.003)+ | -0.254(0.943)+ | -0.269(0.959)+ | 0.058(0.916) | -0.014(1.000) | -0.110(1.001)+ |
| Clinical Frailty Scale, n(%) |  |  |  |  |  |  |
| 1 | 17 (0.3) | 2 (0.1) | 0 (0) | 2 (0.2) | 1 (0.1) | 7 (0.4) |
| 2 | 523 (8.1) | 85 (4.4) | 43 (1.4) | 24 (2.0) | 70 (3.3) | 54 (3.0) |
| 3 | 1,974 (30.5) | 191 (10.0) | 186 (6.1) | 193 (15.3) | 435 (20.5) | 589 (33.1) |
| 4 | 317 (4.9) | 26 (1.4) | 15 (0.5) | 11 (0.9) | 36 (1.7) | 105 (5.9) |
| 5 | 2,386 (36.9) | 645 (33.7) | 730 (23.8) | 345 (27.4) | 442 (20.9) | 347 (19.5) |
| 6 | 889 (13.7) | 613 (32.0) | 1,434 (46.8) | 378 (30.1) | 655 (30.9) | 441 (24.8) |
| 7 | 369 (5.7) | 355 (18.5) | 657 (21.4) | 305 (24.2) | 479 (22.6) | 238 (13.4) |
| ADL, median (IQR) | 0 (0-0) | 0 (0-2)+ | 1 (0-2)+ | 1 (0-2)+ | 1 (0-2)+ | 0 (0-1) |
| IADL, median (IQR) | 1 (0-2) | 3 (1-5) | 4 (2-5) | 2 (1-5)+ | 2 (0-5)+ | 1 (0-4) |
| Number of chronic diseases, median (IQR) | 1 (0-2)+ | 0 (0-1) | 1 (0-1) | 1 (0-2) | 2 (1-3) | 1 (0-2)+ |
| SRH, n (%) |  |  |  |  |  |  |
| Very good | 170 (2.6) | 52 (2.7) | 41 (1.3) | 24 (1.9) | 5 (0.2) | 68 (3.8) |
| Good | 1,771 (27.4) | 625 (32.6) | 662 (21.6) | 412 (32.8) | 207 (9.8) | 565 (31.7) |
| Moderate | 3,037 (46.9) | 878 (45.8) | 1,670 (54.5) | 641 (51.0) | 1,278 (60.3) | 867 (48.7) |
| Bad | 1,334 (20.6) | 315 (16.4) | 628 (20.5) | 175 (13.9) | 579 (27.3) | 256 (14.4) |
| Very bad | 163 (2.5) | 47 (2.5) | 64 (2.1) | 6 (0.5) | 49 (2.3) | 25 (1.4) |
| Energy, n (%) | 1,527 (23.6) | 63 (3.3) | 156 (5.1) | 300 (23.9) | 388 (18.3) | 146 (8.2) |
| Physical activity, n (%) | 936 (14.5) | 227 (11.8) | 259 (8.5) | 65 (5.2) | 182 (8.6) | 95 (5.3) |

+ No significant differences were found between these countries.

Table D. Post hoc comparisons using Bonferroni´s test. Mean differences in cognitive z-score shown by Clinical Frailty Scale Level stratified by sex and countries.

| China | | | | | | | | Ghana | | | | | | | | India | | | | | | | |
| --- | --- | --- | --- | --- | --- | --- | --- | --- | --- | --- | --- | --- | --- | --- | --- | --- | --- | --- | --- | --- | --- | --- | --- |
| Total | | | | | | | | Total | | | | | | | | Total | | | | | | | |
| 1 | 1 | 2 | 3 | 4 | 5 | 6 | 7 | 1 | 1 | 2 | 3 | 4 | 5 | 6 | 7 | 1 | 1 | 2 | 3 | 4 | 5 | 6 | 7 |
| 2 | 0.006 | 1 |  |  |  |  |  | 2 | -0.455 | 1 |  |  |  |  |  | 2 | -0.316 | 1 |  |  |  |  |  |
| 3 | -0.24 | -0.247 | 1 |  |  |  |  | 3 | -0.385 | 0.070 | 1 |  |  |  |  | 3 | -0.671 | -0.355 | 1 |  |  |  |  |
| 4 | -0.51 | -0.515 | -0.268 | 1 |  |  |  | 4 | -0.765 | -0.310 | -0.380 | 1 |  |  |  | 4 | -1.056 | -0.740 | -0.385 | 1 |  |  |  |
| 5 | -0.63 | -0.638 | -0.391 | -0.123 | 1 |  |  | 5 | -0.767 | -0.312 | -0.382 | -0.002 | 1 |  |  | 5 | -0.879 | -0.563 | -0.208 | 0.177 | 1 |  |  |
| 6 | -0.98 | -0.981 | -0.734 | -0.466 | -0.343 | 1 |  | 6 | -1.082 | -0.626 | -0.696 | -0.316 | -0.315 | 1 |  | 6 | -1.126 | -0.810 | -0.455 | -0.070 | -0.247 | 1 |  |
| 7 | -1.42 | -1.423 | -1.176 | -0.908 | -0.785 | -0.442 | 1 | 7 | -1.523 | -1.068 | -1.138 | -0.758 | -0.756 | -0.442 | 1 | 7 | -1.469 | -1.154 | -0.798 | -0.413 | -0.590 | -0.343 | 1 |
| Men | | | | | | | | Men | | | | | | | | Men | | | | | | | |
| 1 | 1 |  |  |  |  |  |  | 1 | 1 |  |  |  |  |  |  | 1 | 1 |  |  |  |  |  |  |
| 2 | 0.096 | 1 |  |  |  |  |  | 2 | -0.295 | 1 |  |  |  |  |  | 2 | -0.285 | 1 |  |  |  |  |  |
| 3 | -0.124 | -0.220 | 1 |  |  |  |  | 3 | -0.260 | 0.035 | 1 |  |  |  |  | 3 | -0.435 | -0.150 | 1 |  |  |  |  |
| 4 | -0.395 | -0.491 | -0.271 | 1 |  |  |  | 4 | -0.545 | -0.250 | -0.285 | 1 |  |  |  | 4 | -0.828 | -0.543 | -0.393 | 1 |  |  |  |
| 5 | -0.522 | -0.617 | -0.398 | -0.127 | 1 |  |  | 5 | -0.577 | -0.282 | -0.317 | -0.032 | 1 |  |  | 5 | -0.651 | -0.366 | -0.216 | 0.177 | 1 |  |  |
| 6 | -0.806 | -0.901 | -0.682 | -0.411 | -0.284 | 1 |  | 6 | -0.805 | -0.510 | -0.545 | -0.260 | -0.228 | 1 |  | 6 | -0.912 | -0.627 | -0.477 | -0.084 | -0.261 | 1 |  |
| 7 | -1.327 | -1.423 | -1.203 | -0.932 | -0.805 | -0.521 | 1 | 7 | -1.276 | -0.981 | -1.016 | -0.731 | -0.699 | -0.471 | 1 | 7 | -1.218 | -0.934 | -0.783 | -0.390 | -0.567 | -0.307 | 1 |
| Women | | | | | | | | Women | | | | | | | | Women | | | | | | | |
| 1 | 1 |  |  |  |  |  |  | 1 | 1 |  |  |  |  |  |  | 1 | 1 |  |  |  |  |  |  |
| 2 | -0.121 | 1 |  |  |  |  |  | 2 | -0.817 | 1 |  |  |  |  |  | 2 |  | 1 |  |  |  |  |  |
| 3 | -0.396 | -0.275 | 1 |  |  |  |  | 3 | -0.630 | 0.187 | 1 |  |  |  |  | 3 |  | -0.687 | 1 |  |  |  |  |
| 4 | -0.644 | -0.523 | -0.249 | 1 |  |  |  | 4 | -1.008 | -0.191 | -0.378 | 1 |  |  |  | 4 |  | -1.072 | -0.384 | 1 |  |  |  |
| 5 | -0.749 | -0.628 | -0.353 | -0.105 | 1 |  |  | 5 | -1.009 | -0.192 | -0.379 | -0.001 | 1 |  |  | 5 |  | -0.752 | -0.065 | 0.320 | 1 |  |  |
| 6 | -1.133 | -1.011 | -0.737 | -0.488 | -0.383 | 1 |  | 6 | -1.327 | -0.511 | -0.698 | -0.319 | -0.319 | 1 |  | 6 |  | -0.876 | -0.189 | 0.196 | -0.124 | 1 |  |
| 7 | -1.519 | -1.398 | -1.123 | -0.875 | -0.770 | -0.387 | 1 | 7 | -1.799 | -0.982 | -1.169 | -0.791 | -0.790 | -0.471 | 1 | 7 |  | -1.245 | -0.558 | -0.173 | -0.493 | -0.369 | 1 |
| Mexico | | | | | | | | Russia | | | | | | | | SouthAfrica | | | | | | | |
| Total | | | | | | | | Total | | | | | | | | Total | | | | | | | |
| 1 | 1 | 2 | 3 | 4 | 5 | 6 | 7 | 1 | 1 | 2 | 3 | 4 | 5 | 6 | 7 | 1 | 1 | 2 | 3 | 4 | 5 | 6 | 7 |
| 2 | 0.075 | 1 |  |  |  |  |  | 2 | 0.606 | 1 |  |  |  |  |  | 2 | 0.067 | 1 |  |  |  |  |  |
| 3 | -0.12 | -0.193 | 1 |  |  |  |  | 3 | 0.172 | -0.434 | 1 |  |  |  |  | 3 | -0.224 | -0.290 | 1 |  |  |  |  |
| 4 | -0.51 | -0.581 | -0.388 | 1 |  |  |  | 4 | -0.299 | -0.905 | -0.471 | 1 |  |  |  | 4 | -0.808 | -0.875 | -0.584 | 1 |  |  |  |
| 5 | -0.3 | -0.371 | -0.178 | 0.210 | 1 |  |  | 5 | -0.141 | -0.747 | -0.312 | 0.159 | 1 |  |  | 5 | -0.791 | -0.858 | -0.567 | 0.017 | 1 |  |  |
| 6 | -0.39 | -0.467 | -0.274 | 0.114 | -0.096 | 1 |  | 6 | -0.476 | -1.082 | -0.648 | -0.177 | -0.336 | 1 |  | 6 | -0.622 | -0.688 | -0.398 | 0.187 | 0.170 | 1 |  |
| 7 | -0.61 | -0.681 | -0.488 | -0.100 | -0.310 | -0.214 | 1 | 7 | -1.006 | -1.612 | -1.178 | -0.707 | -0.865 | -0.530 | 1 | 7 | -0.927 | -0.994 | -0.704 | -0.119 | -0.136 | -0.306 | 1 |
| Men | | | | | | | | Men | | | | | | | | Men | | | | | | | |
| 1 | 1 |  |  |  |  |  |  | 1 | 1 |  |  |  |  |  |  | 1 | 1 |  |  |  |  |  |  |
| 2 | 0.572 | 1 |  |  |  |  |  | 2 | 0.603 | 1 |  |  |  |  |  | 2 | 0.205 | 1 |  |  |  |  |  |
| 3 | 0.212 | -0.360 | 1 |  |  |  |  | 3 | 0.097 | -0.507 | 1 |  |  |  |  | 3 | -0.117 | -0.322 | 1 |  |  |  |  |
| 4 | -0.263 | -0.836 | -0.475 | 1 |  |  |  | 4 | -0.166 | -0.769 | -0.263 | 1 |  |  |  | 4 | -0.622 | -0.826 | -0.504 | 1 |  |  |  |
| 5 | -0.058 | -0.631 | -0.270 | 0.205 | 1 |  |  | 5 | -0.258 | -0.861 | -0.355 | -0.092 | 1 |  |  | 5 | -0.658 | -0.863 | -0.541 | -0.037 | 1 |  |  |
| 6 | -0.184 | -0.756 | -0.396 | 0.079 | -0.125 | 1 |  | 6 | -0.456 | -1.059 | -0.553 | -0.290 | -0.198 | 1 |  | 6 | -0.488 | -0.693 | -0.371 | 0.133 | 0.170 | 1 |  |
| 7 | -0.355 | -0.927 | -0.567 | -0.092 | -0.297 | -0.171 | 1 | 7 | -1.026 | -1.629 | -1.123 | -0.860 | -0.768 | -0.570 | 1 | 7 | -0.740 | -0.945 | -0.623 | -0.119 | -0.082 | -0.252 | 1 |
| Women | | | | | | | | Women | | | | | | | | Women | | | | | | | |
| 1 | 1 |  |  |  |  |  |  | 1 | 1 |  |  |  |  |  |  | 1 | 1 |  |  |  |  |  |  |
| 2 | -0.676 | 1 |  |  |  |  |  | 2 | 0.607 | 1 |  |  |  |  |  | 2 | -0.111 | 1 |  |  |  |  |  |
| 3 | -0.718 | -0.042 | 1 |  |  |  |  | 3 | 0.229 | -0.378 | 1 |  |  |  |  | 3 | -0.326 | -0.215 | 1 |  |  |  |  |
| 4 | -1.002 | -0.326 | -0.284 | 1 |  |  |  | 4 | -0.397 | -1.005 | -0.627 | 1 |  |  |  | 4 | -0.926 | -0.814 | -0.600 | 1 |  |  |  |
| 5 | -0.808 | -0.132 | -0.090 | 0.194 | 1 |  |  | 5 | -0.081 | -0.688 | -0.310 | 0.317 | 1 |  |  | 5 | -0.888 | -0.777 | -0.562 | 0.037 | 1 |  |  |
| 6 | -0.892 | -0.216 | -0.174 | 0.110 | -0.084 | 1 |  | 6 | -0.488 | -1.095 | -0.717 | -0.090 | -0.407 | 1 |  | 6 | -0.725 | -0.613 | -0.399 | 0.201 | 0.163 | 1 |  |
| 7 | -1.127 | -0.450 | -0.408 | -0.124 | -0.318 | -0.234 | 1 | 7 | -1.002 | -1.609 | -1.231 | -0.605 | -0.921 | -0.514 | 1 | 7 | -1.058 | -0.947 | -0.732 | -0.132 | -0.170 | -0.333 | 1 |

Table E. Sex-stratified multinomial regression models for composite cognitive z-score and Clinical Frailty Scale level by country (level 1 as reference)

| CFS level | Men | | | | | |
| --- | --- | --- | --- | --- | --- | --- |
|  | China∫ | Ghana∫ | India ∫ | Mexico ∫ | Russia ∫ | SouthAfrica ∫ |
| 1 | Reference level  RRR ( 95% CI, p-value) | | | | | |
| 2 | 1.01  (0.65-1.56, 0.98) | 0.91 (0.34-2.39, 0.845) | 1.06 (0.45-2.47, 0.899) | 2.81 (0.73-10.78, 0.132) | 4.39 (0.65-29.75, 0.130) | 1.71 (0.73-3.98, 0.216) |
| 3 | 0.85  (0.55-1.31, .456) | 1.08(0.41-2.84, 0.870) | 1.05 (0.45-2.42, 0.912) | 1.86 (0.54-6.39, 0.326) | 2.87 (0.43-19.13, 0.277) | 1.17(0.52-2.60, 0.704) |
| 4 | 0.61  (0.39-0.96, 0.032) | 0.87 (0.30-2.50) | 0.62(0.24-1.59, 0.322) | 1.04 (0.26-4.11, 0.954) | 1.74 (0.25-12.21, 0.579) | 0.72 (0.31-1.71, 0.461) |
| 5 | 0.61  (0.39-0.94, 0.024) | 0.84 (0.2-2.21, 0.729) | 0.90 (0.39-2.07, 0.809) | 1.55 (0.45-5.33, 0.487) | 2.19 (0.33-14.66, .418) | 0.61 (0.27-1.37, 0.231) |
| 6 | 0.52 (0.33-0.81, 0.004) | 0.71 (0.27-1.87, 0.491) | 0.78 (0.34-1.80, 0.566) | 1.28 (0.37-4.39, 0.699) | 1.92 (0.29-12.84, 0.501) | 0.80(0.36-1.80, 0.592) |
| 7 | 0.32(0.20-0.50, <0001) | 0.37 (0.14-0.98, 0.045) | 0.54 (0.23-1.25, 0.151) | 1.26 (0.37-4.34, 0.714) | 1.04(0.15-7.03, 0.966) | 0.66 (0.29-1.51, 0.325) |

| CFS level | Women | | | | | |
| --- | --- | --- | --- | --- | --- | --- |
|  | China∫ | Ghana∫ | India ∫ | Mexico ∫ | Russia ∫ | SouthAfrica ∫ |
| 1 | Reference level  RRR ( 95% CI, p-value) | | | | | |
| 2 | 1.15 (0.63-2.10, 0.644) | 0.45 (0.08-2.65, 0.378) | Reference level | 0.64 (0.08-5.29 , 0.682) | 3.42 (0.32-36.82, 0.311) | 0.75 (0.31-1.82, 0.522) |
| 3 | 0.88 (.048-1.58, 0.660) | 0.62 (0.11-3.60, 0.595) | 0.56 (0.38-0.84, 0.004) | 0.67 (0.09-5.26, 0.706) | 2.47 (0.23-26.20, 0.453) | 0.66 (0.29-1.55, 0.342) |
| 4 | 0.70(0.38-1.28, 0.249) | 0.48 (0.08-3.00, 0.436) | 0.42 (0.20-0.88, 0.022) | 0.45 (0.05-3.93, 0.474) | 1.24 (0.19-21.07, 0.569) | 0.39(0.17-0.94, 0.036) |
| 5 | 0.72 (0.40-1.30, 0.271) | 0.45(0.08-2.61, 0.374) | 0.70 (0.49-1.02, 0.063) | 0.70(0.09-5.43, 0.730) | 1.99 (0.19-21.07, 0.569) | 0.39(0.17-0.91, 0.030) |
| 6 | 0.53 (0.29-0.96, 0.037) | 0.31 (0.05-1.81, 0.193) | 0.67 (0.46-0.97, 0.032) | 0.70(0.09-5.44, 0.732) | 1.48 (0.14-15.73, 0.744) | 0.49 (0.21-1.15, 0.101) |
| 7 | 0.39 (0.21-0.71, 0.002) | 0.16 (0.03-0.95, 0.043) | 0.46 (0.31-0.67, p<0.001) | 0.61 (0.08-4.75, 0.636) | 0.95 (0.09-10.14, 0.  969) | 0.36 (0.15-0.84, 0.018) |

CFS= Clinical Frailty Scale, RRR=Relative Risk Ratio, CI= Confidence Interval

∫ Adjusted for age and education
